# High density terahertz frequency comb produced by coherent synchrotron radiation

**DOI:** 10.1038/ncomms8733

**Published:** 2015-07-20

**Authors:** S. Tammaro, O. Pirali, P. Roy, J.-F. Lampin, G. Ducournau, A. Cuisset, F. Hindle, G. Mouret

**Affiliations:** 1 AILES Beamline, Synchrotron SOLEIL, l'Orme des Merisiers, Saint-Aubin, Gif-sur-Yvette 91192, France; 2 Laboratoire de Physico-Chimie de l'Atmosphère, Université du Littoral Côte d'Opale, EA4493 CNRS, 189A Avenue Maurice Schumann, Dunkerque 59140, France; 3 Institut des Sciences Moléculaires d'Orsay, UMR8214 CNRS—Université Paris-Sud, Bâtiment 210, Orsay 91405, France; 4 Institut d'Electronique de Microélectronique et de Nanotechnologie, UMR8520 CNRS—Université de Lille 1, Avenue Poincaré-Cité Scientifique CS 60069, Villeneuve d'Ascq 59652, France

## Abstract

Frequency combs have enabled significant progress in frequency metrology and high-resolution spectroscopy extending the achievable resolution while increasing the signal-to-noise ratio. In its coherent mode, synchrotron radiation is accepted to provide an intense terahertz continuum covering a wide spectral range from about 0.1 to 1 THz. Using a dedicated heterodyne receiver, we reveal the purely discrete nature of this emission. A phase relationship between the light pulses leads to a powerful frequency comb spanning over one decade in frequency. The comb has a mode spacing of 846 kHz, a linewidth of about 200 Hz, a fractional precision of about 2 × 10^−10^ and no frequency offset. The unprecedented potential of the comb for high-resolution spectroscopy is demonstrated by the accurate determination of pure rotation transitions of acetonitrile.

## Abstract (web summary)

There is considerable interest in generating broadband frequency combs at terahertz frequencies. Here, Tammaro *et al.* achieve this using coherent synchrotron radiation where the electron bunches emit quasi-synchronous terahertz pulses with high power, broad frequency, zero frequency offset, and high density.

## Graphical abstract

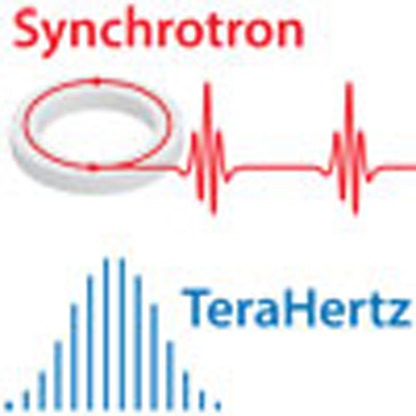

Frequency combs (FC) have radically changed the landscape of optical instrumentation containing a vast number of narrow optical modes at a series of well-defined frequencies, which may be used as a highly accurate ruler over its spectral range. Initially developed in the visible and near-infrared spectral regions^1^, the use of FC has been expanded to the mid-infrared^2^, extreme ultra-violet^3^, X-ray^4^ regions and significant effort is currently being dedicated to the generation of FC at THz frequencies. One solution based on converting a stabilized optical FC using a photoconductive terahertz emitter remains hampered by the low available THz power^5^,^6^. Another approach is based on active mode locked THz quantum-cascade-lasers providing intense FC over a relatively limited spectral range^7^,^8^,^9^.

As an alternative to laser based experiments and electronic generation, broadband THz radiation emitted from short electron bunches is now used for high-resolution spectroscopy and spectro-microscopy applications. In the so-called low-α mode, intense THz power is obtained by reducing the electron bunch length to produce phase coherent synchrotron radiation (CSR)^10^. Following the first demonstration, such radiation has been developed in several synchrotron facilities over the world. It has provided unprecedented power in the region 0.1−1 THz, up to 4 orders of magnitude brighter than standard incoherent synchrotron radiation^10^,^11^. Super-Radiance effects obtained from bunch-to-bunch interference^12^ further enhance specific frequencies (*1/T*_bunch_ where *T*_bunch_ is the time interval between successive bunches) of the THz CSR.

In this work, using a dedicated high-performance sub-THz heterodyne receiver (see Methods), we reveal that THz CSR is an intense, stable (in both frequency and amplitude), dense zero offset FC covering a wide spectral range 0.1–1 THz with no continuum background.

## Results

### Spectral characterization of CSR

**Figure 1:**
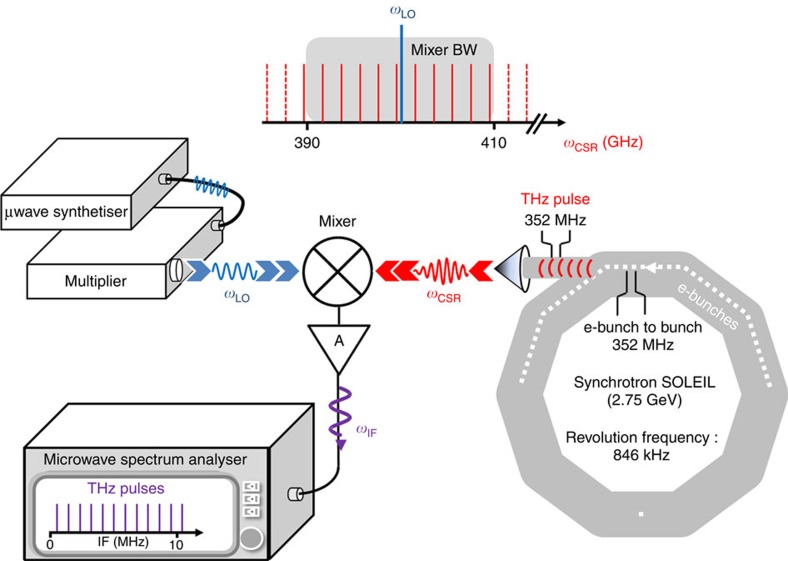
CSR heterodyne detection schematic. Short bunches of relativistic electrons circulating in the SOLEIL storage ring emit CSR in the 0.1–1 THz range. The bunch-to-bunch frequency 1/*T*_bunch_ is 352 MHz, while the storage ring revolution frequency is 846 kHz (see Methods). The THz CSR pulses are mixed with the monochromatic radiation (*f*_LO_) from a local oscillator (microwave synthesizer and multiplier) creating an electronic signal at the intermediate frequency (IF): *f*_IF_*=*|*f*_LO_*−f*_CSR_| within the mixer bandwidth. The resulting signal is amplified by the IF amplifier A and analysed using a microwave spectrum analyser. The mixing produces a frequency downconversion of *f*_CSR_ into the microwave range, and an aliasing of the spectrum due to the superposition of the upper (*f*_CSR_>*f*_LO_) and the lower (*f*_CSR_<*f*_LO_) frequency bands.

**Figure 2:**
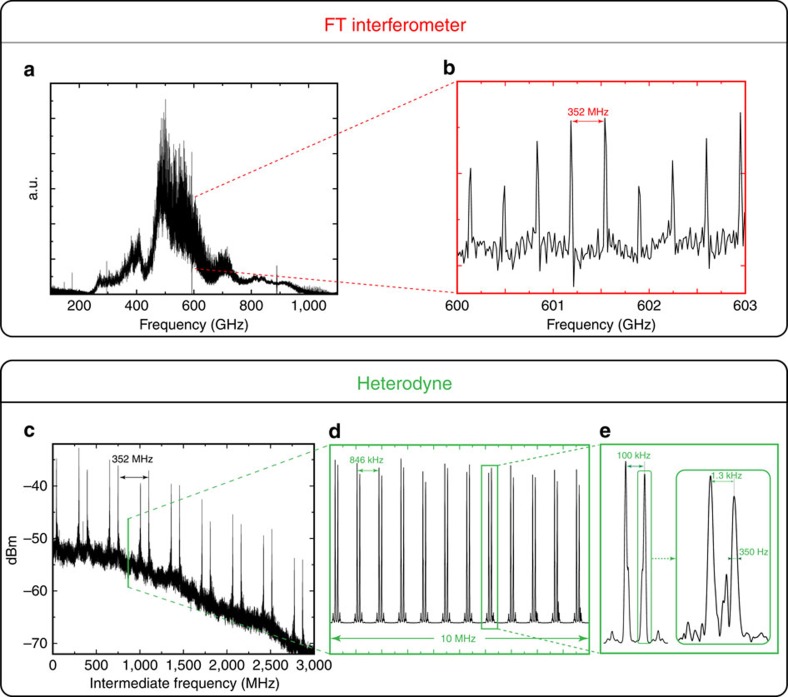
CSR FC structure recorded using the FTIR interferometer and heterodyne receiver. All amplitude scales are linear unless stated otherwise. Heterodyne frequency scales are established as a function of the intermediate frequency. (**a**) Full range of the CSR measured by the FTIR interferometer with a resolution of 30 MHz; the low-frequency response is limited by the optical elements of the instrument. (**b**) Zoom of 3 GHz revealing the 352 MHz FC corresponding to 1/*T*_bunch_. (**c**) Heterodyne spectrum obtained by mixing CSR with *f*_LO_=200 GHz. The FC at 352 MHz is also easily distinguished. (**d**) The second FC composed of sharp teeth regularly spaced by 846 kHz is revealed by an expanded frequency scale. This second comb is produced by the very stable revolution period of the electron bunches in the storage ring (1.18 μs). (**e**) A zoom of a given dual comb structure caused by the aliasing of the upper and lower frequency bands. (**e**), inset: the excellent resolving power of the frequency scale allows the detailed examination of a single tooth, highlighting the ultrafine splitting due to low-frequency instabilities of the electron bunches.

In this work, the CSR spectral structure has been probed by a Bruker Fourier Transform Infrared (FTIR) instrument with an ultimate resolution of 30 MHz, as well as by the proposed heterodyne receiver shown in Fig. 1. The receiver, capable of operation in a large spectral region with a bandwidth of about 10 GHz, probes the synchrotron radiation with unprecedented spectral resolution, high signal-to-noise ratio and excellent frequency accuracy. Using both spectrometers, we are able to detect the fine spectral features of the CSR. In particular, we identified two main ultra-stable FC sequences in both regions studied (200±10 GHz and 400±10 GHz). The FC sequences are illustrated in Fig. 2 with a progressively finer frequency scale. The upper panel of this figure shows the CSR spectrum as recorded by the FTIR spectrometer at the highest possible spectral resolution (maximum optical path difference of 8.82 m, corresponding to a resolution of 30 MHz). The full CSR range convoluted by FTIR response is shown in Fig. 2a. A detailed view ∼600 GHz reveals a 352 MHz FC superimposed on the broad THz emission, Fig. 2b, as previously observed in other facilities^12^. The lower panel shows the heterodyne analysis of the CSR. Figure 2c shows 3 GHz of heterodyne spectrum at 200 GHz, in which the 352 MHz FC originating from the bunch-to-bunch repetition is also clearly resolved. This FC is the signature of a degree of coherence among the electron bunches^13^ and is designated as super-radiance emission. Expanding the frequency scale further reveals a second FC composed of sharp teeth regularly spaced by 846 kHz, Fig. 2d. This comb is indeed related to the very stable revolution period of the electron bunches in the storage ring (1.18 μs). It produces a spectrally dense THz FC with >10^6^ components covering the THz range from 0.1 to 1 THz. The observed structure as shown in Fig. 2d is composed of the lower and upper frequency band contributions as expected for heterodyne detection technique. Aliasing of the FC results in the observation of a doubled FC structure whose frequency separation is dependent on the local oscillator (LO) frequency. Shifting the LO frequency allowed the two components to be distinguished, here a separation of 100 kHz was selected, Fig. 2e. The detailed examination of any individual tooth, Fig. 2e inset, indicates an even narrower structure: typically, two components separated by 1.3 kHz, each with a full width at half maximum (FWHM) systematically <500 Hz. The substructure is interpreted as a consequence of the kHz range amplitude modulation caused by the low-frequency instabilities of the electron bunches^14^.

### Power measurement

The heterodyne analysis allowed the power of an individual CSR line ∼400 GHz to be estimated at −77 dBm (that is, 20 pW) (see Methods). The CSR spectral power density is 3 orders of magnitude greater than can be achieved from a classical dipolar antenna used in a context of THz time domain spectroscopy^5^. The dynamic range of the amplitude of the FC teeth is systematically in excess of 35 dB with the strongest lines exhibiting over 70 dB.

### Offset determination

In general, FC modes are described as multiples of a reference frequency (*f*_R_) shifted by an offset (*f*_0_): *f*_FC_*=n × f*_R_*+f*_0_, where *n* is an integer. The offset frequency was assessed by measuring the revolution frequency with a counter (CNT 90, Pendulum). Over a period of 20 s, the revolution frequency is determined with a typical uncertainty of 30 μ Hz. The comb mode centre frequencies were determined by the spectral analysis of the intermediate frequency (IF) signal. In excess of 50 modes were examined, each being found to be an exact multiple of the revolution frequency with a confidence interval of 1 Hz. Unlike optical FC, the CSR FC is therefore considered to be offset free.

### Pure rotational spectroscopy of acetonitrile

**Figure 3:**
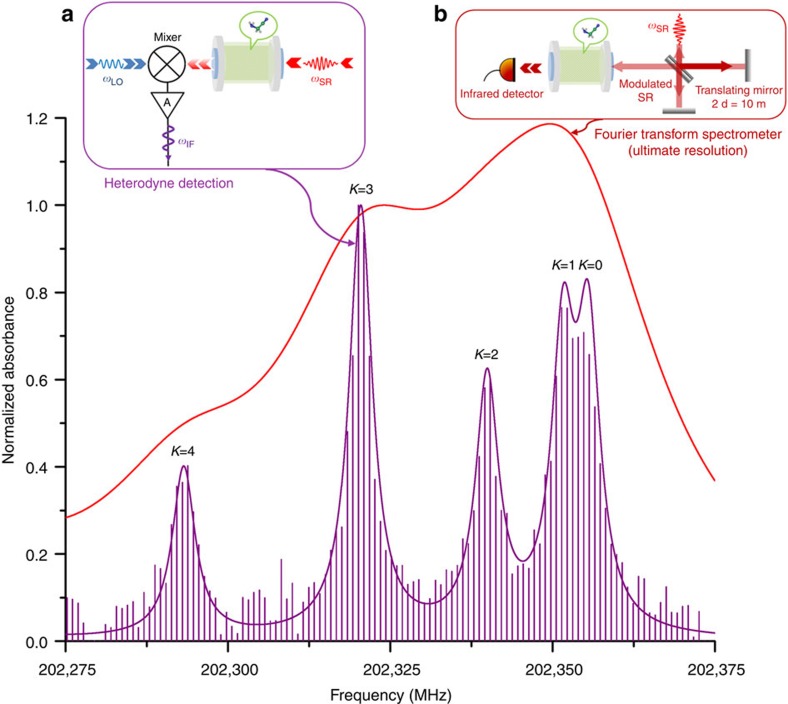
Normalized absorbance of acetonitrile. Normalized absorbance versus frequency showing the *K* structure of the pure rotational R(10) transition of acetonitrile recorded with the heterodyne receiver, *f*_LO_=202.0 GHz (violet vertical lines). The gas pressure was set to 50 μbar, the measurement and background IF spectra are processed identically to isolate the upper frequency band FC only (*f*_CSR_>*f*_LO_). The violet solid line is the result of the fit of the 5 individual lines using Voigt profiles (see Methods), while the red curve corresponds to a simulation at the maximum possible resolution (30 MHz) of commercially available Fourier Transform (FT) spectrometers. The instrument configurations are schematically represented in the insets. Inset (**a**): heterodyne measurement, the synchrotron radiation transmitted through a cell filled with gas is incident on a mixer simultaneously with the radiation from a local oscillator. (**b**), inset: absorbance measurement based on FT method, the interferometre-modulated radiation passes through a gas-containing cell before arriving at the detector.

We recorded the absorption spectrum of acetonitrile (CH_3_CN) as the *K* series of this symmetric top molecule is particularly well suited to test the frequency accuracy and the dynamic range of the CSR FC. A 1.3-m-long absorption gas cell was inserted into the CSR path. Figure 3 shows a small section of the acetonitrile absorption spectrum obtained in 65 s using the CSR FC with the LO at 202 GHz. The FC gives rise to a discrete spectrum built from the harmonics of the repetition rate of 846 kHz (violet vertical lines). The complete *K* structure of the R(10) transition of CH_3_CN is clearly resolved (violet profile, as explained in the associated content). This is in strong contrast to the simulated spectrum corresponding to the 30 MHz resolution of the IFS125 interferometre (red profile), only displaying a broad unresolved feature. In particular, the THz FC allows the doublet *K*=0 and *K*=1 separated by 3.9 MHz (1.3 × 10^−4^ cm^−1^) to be resolved. The fit of the five individual absorption lines with Voigt profiles provides their central frequencies, which are in excellent agreement with reference data (r.m.s<80 kHz) (see Methods). The accuracy obtained is comparable with frequency multiplication techniques currently employed in this frequency range.

## Discussion

The measurements described in the previous section shows clearly the discrete nature of the CSR. Moreover, the relatively high power together with the frequency stability of this source allows for room temperature heterodyne fast detection. Such results demonstrate that the phase relation between the emissions from bunches exists and is preserved over a long time to form a dense FC. In this sense, in the low alpha configuration, the operational mode of the synchrotron can be likened to ultrashort laser pulses where the first repetition rate is determined by the length of the ring resonator, and a second being defined by the bunch-to-bunch spacing. It is noteworthy that by using the same detection scheme, no discrete spectral components and no FC have been observed in standard synchrotron mode proving that for longer bunches of electrons there is no phase coherence between subsequent pulses, preventing the formation of a dense FC.

As shown by the quality of the THz acetonitrile absorption spectrum, the CSR FC is particularly suited to high-resolution gas phase THz spectroscopy as the mode spacing of 846 kHz is the same order of magnitude of the Doppler-limited linewidths of a typical rotational transition of a light polar compound at room temperature. This is not the case for many laser-generated THz combs with mode spacing typically >100 MHz, requiring complex interleaving strategies to be adopted to obtain Doppler-limited rotational spectra^6^,^15^. Indeed the CSR FC composed of powerful, fine modes over an extended spectral range (0.1–1 THz) should outperform FTIR instruments in terms of resolution and analysis times. In comparison to electronic sources that offer an excellent resolution, the advantage of CSR FC is the spectral coverage of one decade, whereas a frequency multiplier may offer a tunability of ∼20%, numerous multiplier combinations are required to cover an octave of frequency. The excellent resolving power, frequency metrology and high dynamic range of the CSR FC are demonstrated by the high-resolution spectroscopy measurements undertaken using the heterodyne receiver initially constructed for the spectral analysis.

To harness the full potential of this FC, a solution must be sought to simultaneously access its entire frequency band (that is, over more than one decade of frequency). Mixing the CSR FC with an optical comb in a suitable photoconductor should yield the required information via a dual comb spectroscopy approach unlocking the advantages of the CSR^15^.

The CSR FC characteristics revealed here clearly prompt the development of receivers for ultra-high resolution over broader frequency ranges. This is an enabling approach for new high-resolution THz time-resolved experiments. Among highly desirable new applications are ultrafast monitoring of reaction kinetics, ultra-high resolution atomic and molecular spectroscopy^16^, and spatio-temporal dynamics of relativistic electron bunches.

## Methods

### Coherent synchrotron radiation

The AILES beamline collecting the edge emission^17^ from the 2.75 GeV, third generation Synchrotron Radiation source SOLEIL, has an extended programme of research with synchrotron radiation in the THz spectral range. In particular, a special attention is paid into increasing the flux while maintaining source stability through optimization of CSR^11^. The SOLEIL magnet lattice allows for the storage ring low alpha operation appropriate for the generation of the CSR in the THz spectral range. For this operation, relativistic electrons entering the dipole magnetic field are known to emit coherently at wavelengths comparable with or larger than the electron bunch length. In presence of a storage ring vacuum chamber, the coherent spontaneous emission can be extracted for wavelengths smaller than the cut-off wavelength value of the vacuum chamber, acting as a waveguide. The principle consists in providing conditions for stable electron dynamics with the smallest possible bunch length. In storage rings, this typically requires a low value of the momentum compaction factor and a smaller electron bunch length. The complete sequences of intense THz FC combs were observed in the low α coherent mode characterized by a 4.8 ps r.m.s bunch length and corresponding to a compaction factor *α*=*α*_0_/25, with *α*_0_=1.7 × 10^−5^ being the nominal value^14^. The ring was injected with 208 electron bunches distributed on one half of the SOLEIL ring and by one isolated bunch in the centre of the other half. In this operation mode the total current was about 16 mA with periodic refilling in top-up mode. Two principal periods characterize the temporal radiation distribution: (i) the 208 bunches produce a 2.8 ns periodic pulsed radiation train and (ii) the revolution period in the ring is about 1.18 μs.

### Heterodyne detection

To investigate the spectral structure of the THz CSR, we constructed a high sensitivity coherent electronic receiver^18^. Heterodyne detection is very convenient for high-resolution spectral analysis with a high dynamic range. The operation is based on a frequency translation of the analysed spectra by electrical mixing between a known reference frequency (LO) and the signal to be analysed. The mixing element is a Schottky-based sub-harmonic-mixer (SHM), fed by the LO at mW level (continuous wave mode), this power being required to operate the Schottky device in a non-linear regime. Two different SHM mixers WR5.1 (140–220 GHz) and WR2.2 (325–500 GHz) are used for operation at 200 GHz and 400 GHz, respectively. The LO is generated by an electronic frequency multiplication chain driven by a Rohde and Schwarz SMF100A synthesizer. This provides equivalent frequency multiplication factors of × 12 and × 24, respectively. The IF signal is amplified with two base-band amplifiers, providing a total gain of 45 dB over a bandwidth of 500 MHz. The downconverted CSR signal is analysed by a 3.6 GHz Agilent MXA Signal Analyzer N9020A.

### Power measurements

From IF signal spectral analysis, the individual CSR lines located around 400 GHz display a typical signal strength of −40 dBm. Taking into account the 45 dB IF gain and a 8 dB conversion loss of SHM, the corresponding power of CSR lines should be around −77 dBm (20 pW). The integrated THz power of the CSR was measured to be 60.5 μW by a Gentec THz 5B-BL-DZ pyroelectric detector working at room temperature. The beam was filtered with a black polyethylene film to avoid any effect of visible or mid-infrared radiation. The ratio of the integrated power to the power of one line is then equal to about 3 × 10^6^. This gives a rough estimation of the number of lines assuming that all lines of the FC have the same intensity. This value is compatible with the fact that in a 1 THz range there should be about 1.2 × 10^6^ lines separated by 846 kHz. The spectral power density is hence calculated to be 23.6 nW GHz^−1^.

### Spectroscopic analysis on acetonitrile

The IF spectra are processed to isolate the lower and upper frequency band contributions. Once all the peaks in the spectra have been identified, the FC spacing of 846 kHz is used to distinguish each FC component. In Fig. 3, the normalized absorbance of CH_3_CN measured with the THz FC was simulated with Voigt profiles. The Doppler linewidth (Gaussian contributions) of the five components has been fixed to a calculated value at room temperature of 0.4 MHz (FWHM). The centre frequencies and the collisional linewidths (Lorentzian contributions) were fitted for *K*=0–5 components of the R(10) transitions. In Table 1, the fitted frequencies *f*_fitted_ are compared with the frequencies *f*_calc_ listed in the JPL database^19^ and calculated from the latest combined fit reported by Müller *et al.*^20^. The r.m.s determined from the (*f*_fitted_−*f*_calc_) values is evaluated to be to 79 kHz. The collisional linewidths were fitted to 3.4 MHz (FWHM) for the five components (no *K* dependence of the R(10) self-broadening was observed). At a pressure of 50 μbar a collisional linewidth of ∼4.5 MHz (FWHM) may be estimated for the R(10) transitions from CH_3_CN self-broadening measurements^21^,^22^

## Additional information

**How to cite this article:** Tammaro, S. *et al.* High density THz frequency comb produced by coherent synchrotron radiation. *Nat. Commun.* 6:7733 doi: 10.1038/ncomms8733 (2015).

## Figures and Tables

**Table 1:** fitted frequencies compared with the frequencies listed in the JPL database^19^.

***K***	***f***_**fitted**_ **(MHz)**	***f***_**calc**_ **(MHz)**	***f***_**fitted**_−***f***_**calc**_ **(MHz)**	**100 × (*****f***_**fitted**_**−*****f***_**calc**_**)/*****f***_**calc**_
0	202,355.39	202,355.51	−0.12	5.93E**−**05
1	202,351.73	202,351.61	0.12	5.93E**−**05
2	202,339.88	202,339.92	−0.04	1.97E**−**05
3	202,320.42	202,320.44	−0.02	9.89E**−**06
4	202,293.19	202,293.18	0.01	4.94E**−**06
